# A Non-Surgical Treatise on Diseases of the Prostate Gland and Adnexa

**Published:** 1904-03

**Authors:** 


A NON-SURGICAL TREATISE ON DISEASES OF THE PROSTATE GLAND AND ADNEXA. By George Whitfield Overall, A. B., M. D., formerly Professor of Physiology in the Memphis Hospital Medical College. Price not given. Published by Marsh & Grant Company, Chicago.

The author early takes his stand against the indiscriminate and reckless use of the knife in the treatment of prostatic diseases. That there are some cases in which surgery must be resorted to, he does not deny, but that these cases can be reduced to a minimum by the thorough and scientific application of medicines, electrolysis and cataphoresis his large experience has full demonstrated.

The work is of convenient size, clear outline, thoroughly practical, and will fill a conspicuous place in routine use.

